# Is There a Need to Integrate Human Thermal Models with Weather Forecasts to Predict Thermal Stress?

**DOI:** 10.3390/ijerph16224586

**Published:** 2019-11-19

**Authors:** Jakob Petersson, Kalev Kuklane, Chuansi Gao

**Affiliations:** 1 Thermal Environment Laboratory, Division of Ergonomics and Aerosol Technology, Department of Design Sciences, Faculty of Engineering, Lund University, 22362 Lund, Sweden; chuansi.gao@design.lth.se; 2 Institute for Safety (IFV), 2701 AC Zoetermeer, The Netherlands; Kalev.Kuklane@ifv.nl

**Keywords:** heat stress, cold stress, human thermal models, meteorological forecast, thermal stress warning, heat wave, cold spell

## Abstract

More and more people will experience thermal stress in the future as the global temperature is increasing at an alarming rate and the risk for extreme weather events is growing. The increased exposure to extreme weather events poses a challenge for societies around the world. This literature review investigates the feasibility of making advanced human thermal models in connection with meteorological data publicly available for more versatile practices and a wider population. By providing society and individuals with personalized heat and cold stress warnings, coping advice and educational purposes, the risks of thermal stress can effectively be reduced. One interesting approach is to use weather station data as input for the wet bulb globe temperature heat stress index, human heat balance models, and wind chill index to assess heat and cold stress. This review explores the advantages and challenges of this approach for the ongoing EU project ClimApp where more advanced models may provide society with warnings on an individual basis for different thermal environments such as tropical heat or polar cold. The biggest challenges identified are properly assessing mean radiant temperature, microclimate weather data availability, integration and continuity of different thermal models, and further model validation for vulnerable groups.

## 1. Introduction

Modern societies are challenged by extreme weather events accompanied by thermal stress related to heat and cold, and this challenge is expected to increase with future climate changes [1]. The increasing heat exposure affects outdoor workers [2] and other vulnerable groups such as the elderly, children, and people with cardiovascular and respiratory diseases in particular [3,4,5,6]. Cold stress remains a problem in many countries, both through harsh winter weather [7,8] but also in artificial cold environments such as cold warehouses [9,10].

Mapping thermal stress by taking the thermal climate into consideration is very important for both health and wellbeing. As a result of thermal stress, body thermal strain will cause discomfort, heat, and cold related illnesses, decreased performance and increased risk of accidents. When under severe thermal strain, physical work capacity and cognitive functions decrease or even become impaired and life may become threatened. Previous studies have not only identified risks with thermal environments, but also quantified the related problems in terms of productivity loss and mortality [11,12].

Today, many models to predict thermal stress are available, but these are developed for occupational conditions and need further validation specifically to be used for vulnerable groups. Using models to predict heat stress in a precautious manner have reduced risks for morbidity and mortality in industrial, military, sports, and leisure activities [13].

However, these internationally established thermal models with open source codes in the form of ISO standards are not widely accessible, neither are they used in connection with meteorological data to serve occupational and public health. Therefore, there is a need to link these thermal models to meteorological data to predict health risks in a changing climate [14].

To assess the thermal stress, six parameters needs to be covered: four thermal climate factors consisting of air temperature, humidity, air velocity, and heat radiation, and two individual factors that are metabolic rate and clothing [15]. As a way of simplifying heat stress prediction, Lemke and Kjellstrom investigated the possibility to predict heat stress using local weather forecast data [16]. They focused on the wet bulb globe temperature (WBGT) index, which is a globally used screening tool for heat stress [17].

In addition to WBGT used to assess heat stress in connection with meteorological data, other heat indices were discussed by Burgstall et al. (2019) to investigate if the heat warning system in Switzerland could be improved. The following indices were compared: WBGT, WBGT in shade, WBGT in sun, simplified WBGT, apparent temperature, effective temperature, humidity index and discomfort index. They simulated the number of warnings that matched for each index with their current heat index warning system. Their investigation concluded that the desired index depends greatly on the dominating variable and that there is no index to properly catch them all, but that an index putting great weight on both temperature and humidity is at advantage for human heat stress research. One index that matched rather poorly with the current heat index was WBGT, indicating that either the prediction of WBGT was poor or that the current system is skewed as it does not properly address radiation [18]. A state-of-the-art review performed by Casanueva et al. (2019) investigated the heat-health warning systems and action plans for 16 countries in Europe and references to the global scale, where the most commonly used warning system make use of the maximum temperature of the day whereas few use heat stress indices that take human heat balance into account [19]. The use of environmental indices can provide good screening tools for heat warnings but for every disregarded parameter there is a loss of information. Staiger et al. (2019) suggested the use of universal thermal climate index (UTCI), perceived temperature, physiological equivalent temperature, and effective temperature [20]. These indices take metabolic rate and clothing insulation into account, however they are often predetermined and therefore limited from the individual perspective. The EU project HEAT-SHIELD has developed a platform to apply the heat stress index WBGT with meteorological forecast data to provide a web-based heat-health warning system across Europe with the focus on occupational settings where outdoor workers are the target group. The system predicts the risk based on daily maximum WBGT for long-term forecast (46 days) and aims to provide this service free of charge and valid all over Europe [21].

Building on Lemke and Kjellstrom’s (2012) approach and the proposal to link ISO standards to climate change by Parsons (2013) [14], this review investigates the feasibility of integrating local weather forecast data into multiple thermal models and indices for a wide range of extreme weather conditions (approx. −50 to +50 °C). This review aims to explore the possibility and challenges to integrate meteorological data into human thermal models that are openly available as international standards to provide more precise climate service tools for individuals and society to cope with thermal stress caused by climate change.

## 2. Method

Initially, the pearl picking method was applied. The article “Calculating workplace WBGT from meteorological data: a tool for climate change assessment” by Lemke and Kjellstrom [16] served as the first step to the review to track other key articles. Information retrieval was also performed through Web of Science/Science Citation Index (SCI), PubMed, Scopus, Google Scholar, etc. Key words were used to search for relevant published articles in peer-reviewed journals.

Key words used were: heat stress, occupational heat stress, heat exposure, climate change, climate projection, human thermal model, urban heat island effect, cold stress, thermal stress, thermal climate, heat related mortality, cold related mortality. weather forecast, extreme weather events, climate service, WBGT, PHS, TWL, IREQ, wind chill temperature, and PMV.

The criteria for selection were that the thermal model or index must be readily available with open source code and accepted as an international standard.

## 3. Thermal Exposure and Health Risks in a Changing Climate

### 3.1. Heat Stress

With global warming, it is expected that billions of people living in tropical countries will experience increased heat exposure including increased mean and extreme temperatures and this will most likely affect poor people with laboring occupations in particular [2]. Other parts of the world that do not normally suffer from heat stress will face new challenges. Some countries are more vulnerable to thermal exposure and it is predicted that an already reduced global work capacity during peak temperature months will continue to decrease with a reduction of 80% by 2050 [22]. Kjellstrom et al. (2018) predicts a greater loss of productivity in the tropical and sub-tropical areas than the rest of the world but southern Europe and United States are also affected [23]. Forzieri et al. (2017) analyzed the risk of mortality of the European population and concluded that weather-related disasters could affect two-thirds of the population annually by 2100 and cause 50 times more deaths compared with that of today [24].

The Intergovernmental Panel on Climate Change (IPCC) has predicted that the amount of hot days and nights will increase which will make extreme events more common and heat stress relief less available [1]. A high daily minimum air temperature has been shown to have significant negative effects on sleep [25,26]. Mora et al. (2017) found that around 30% of the world’s population is currently exposed to extreme heat for at least 20 days every year [27]. A few studies have investigated the different projections leading to a 1.5 °C warming and a 2 °C warming, for Europe [23], Asia [28], Northern America [29], and Africa the patterns are very similar for both projections but the intensity is greater in each continent in the 2 °C projection [30]. The probability for a hot extreme in the 2 °C warming is expected to be twice as frequent compared to that in the 1.5 °C warming [31]. The increasing ocean surface water temperature will force further water evaporation to the air, increasing the absolute humidity in the air [32]. As a result, this will affect the sweat evaporation capacity for exposed individuals. With increasing water content in the air it is expected that the amount of precipitation and corresponding clouds will increase [33] which may have a cooling effect on humans.

A study in the Netherlands found that the elderly was the most vulnerable group to the extreme heat and that the heat increased the respiratory mortality rate [34]. Similar results were found in Finland where the heat-related mortality was linked with cardiovascular and respiratory conditions where most of the deceased were elderly [35]. The increase of cardiovascular and respiratory mortality for elderly in heat has been proposed to be attributed to high ozone and PM_10_ levels by several studies while the underlying mechanisms are not fully understood and the relationship needs further investigation [36,37,38,39].

Miller et al. (2011) investigated the concept of self-pacing as a natural preventive mechanism to reduce heat stress when possible [40]. A potential consequence of self-pacing is a loss of work capacity which in turn lowers the productivity [2,23,41]. One approach is to use the thermal work limit, an index that estimates the maximally allowed metabolic rate for a set environment. Healthy and educated staff members are allowed to self-pace below this metabolic rate rather than measuring the individual’s metabolic rate [42]. This may be advantageous for healthy and educated staff but a risk for inexperienced or unfit individuals.

### 3.2. Cold Stress

Heat stress is receiving a lot of coverage in media as global temperature increases and a large part of the global population experience exclusively heat stress, however mortality is higher in winter than in summer in temperate climate [43,44]. Excess morbidity and mortality is attributed to a varied winter weather which is forecasted by climate models even though the global mean temperature is expected to rise [45]. In projections, mortality is still higher in cold than in heat in the UK in 2080 and, elderly people are the most vulnerable [46]. The cold mortality is ten-times higher than heat mortality in Finland, this is attributed to cerebral vascular diseases, coronary heart diseases, and respiratory diseases. Most of the deceased in the cold are the elderly, similar results were found in the Netherlands [34]. Studies in the United States note that the gross mortality is higher during winter months compared to summer months [3,47].

A study covering European cities stress that the cold is a problem all across Europe and that the cold stress effect is higher in the warmer southern cities. Increasing frequencies of respiratory and cardiovascular diseases were positively correlated with decreasing temperatures where the elderly were identified to be particularly at risk [48]. A similar study in China concluded that the effects of cold-affected mortality in the southern cities and effects of heat induced mortality in northern cities, a probable reason is that there is a widespread central heating system in the north to protect against the more common cold climate and air conditioning in the southern cities to alleviate the heat [49].

Certain job positions force the worker to be in contact with cool or cold environments not only outside with exposure to harsh weather conditions but also in cool warehouses and food processing industries. In order to reduce the risk of tissue and body cooling, protective clothing is used to protect the body from heat losses [7,9,50,51]. An analysis by Oliveira et al. (2008) investigated 32 different industrial sites and found that a third of the workers were consistently exposed to cold environments wearing insufficient clothing [52]. In a field study it was noted that the provided work clothing was not insulated enough, some workers also removed their gloves to perform fine tasks [10]. Austad et al. (2018) concluded the need of a decision-support system based on individual data to support cold stress protection [53].

### 3.3. Indoor and Urban Enhancement

In 1950, 30% of the population lived in urban environments. It is projected that in 2030, 60% of the global population will live in cities and on some continents as much as 80–85% [54]. The urban heat island (UHI) has been investigated in many cities. In Shanghai it was found that UHI has a profound effect on human health, by enhancing the intensity of heat waves. It was noted to have the direct effect in city center compared to suburban locales [55]. The UHI is affected by the urban coverage which also affects microclimates, another aspect is the city planning which can lower the temperature with the focus on advection [56]. Urban city planning affects the indoor temperature with factors such as housing density, types of structures, unpaved areas, building arrangement, and urban green area. The most profound contrast between urban and rural settings is during nighttime when the radiation lag is still keeping the urban area warm [57]. The amount of green spaces and vegetation is negatively correlated with the indoor air temperature whereas the storey number is positively correlated [58].

Little is known about the relationship between indoor and outdoor temperatures [59]. A comprehensive literature review on the subject stressed that even though we spend as much as 90% of our everyday life inside, most heat-health warning systems are based on outdoor measurements [60]. Important measures to mitigate indoor heat stress are to install different kinds of shading devices, proper clothing, ventilation, fans, air conditioners powered by renewable power, and phase change materials incorporated into the building material to act as a thermal buffer [60]. A study investigating the indoor climate shows that productivity decreases with 2% for every centigrade increase between 25–32 °C [61].

Gronlund et al. (2018) pointed out that since individuals tend to spend more time indoors during hot or cold weather, they are further exposed to indoor health risks. Listed risks are allergens, combustion products, and volatile organic compounds when adequate ventilation is not provided [44]. As previously mentioned, the elderly are vulnerable to thermal stress [6]. An epidemiological study in Canada noted that older patients that required emergency care tended to stay in warmer housing [62]. A study in Japan show seasonal trends where the physical performance deteriorates for elderly with colder indoor temperature, the overall physical performance was worse for people living in colder houses [63].

## 4. Human Thermal Models and Indices

Occupational thermal stress is a concept established to measure the thermal stress that workers experience during their workday. To comprehensively assess human thermal stress, six basic factors need to be included:

- Air temperature
- Humidity
- Air velocity
- Radiant temperature
- Metabolic rate
- Clothing

Today there is a range of human thermal models and indices to estimate thermal stress available for different scenarios, each with specific strengths and limitations, as well as levels of complexity and simplicity in terms of inputs, algorithms, interpretations, and applications of outputs.

**Figure 1:**
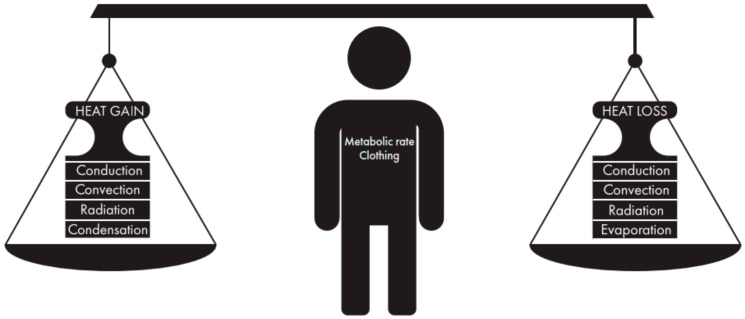
Human body heat balance is calculated by metabolic rate and the heat exchange by radiation, convection, evaporation, and conduction. The heat balance is the result of the positive fluxes and negative fluxes and will be influenced by individual factors and clothing worn.

For the human cells to function properly, the human body regulates the core temperature to be near 37 °C. To maintain this core temperature, the body generates heat through metabolic rate (M) that is partly used to perform mechanical work (W) and mostly used to balance the heat exchanges through evaporation (E), radiation (R), convection (C), and conduction (K) as visualized in Figure 1 [15]. The conceptual heat balance equation is defined as

M − W = E + R + C + K + S

where S is the body heat storage [15]. Increasing or decreasing the core and skin temperatures causes thermal discomfort, heat strain or cold strain, where further heat or cold exposure will put the organs at risk [15] and makes it harder to keep up with production targets during work [2], and eventually lead to heat- or cold-induced illnesses. Cold-related problems related to health and performance start below normal indoor temperature of 20 °C [8]. When core temperatures decrease with a few degrees, cognitive functions become impaired followed by confusion and unconsciousness [64]. Manual work and finger dexterity will deteriorate when the skin temperature decreases to 14 °C [65]. A critical ambient temperature threshold is 35 °C in the heat, above this threshold people who are performing heavy labour are increasingly likely to suffer from heat strain [15] since the skin is warmed and not cooled by convection and radiation. Sweat evaporation and small part of respiratory evaporation is the only avenue to dissipate heat from the body to the environment.

### 4.1. Heat Stress Index and Model

#### 4.1.1. Wet Bulb Globe Temperature (ISO 7243)

The most commonly used heat stress index today is the Wet Bulb Globe Temperature (WBGT) index developed by the US army [17], where heat illness is accredited to a combination of high temperatures, high humidity, and solar radiation, and low air velocity. WBGT is measured by using three different temperatures; the natural wet bulb temperature (Tnw), the 150 mm black globe temperature (Tg), and the air temperature (Ta). The biggest hurdle for Lemke and Kjellstrom’s approach is that variables used for heat stress assessment are not readily available from the conventional weather forecast data. For example, Tnw and Tg are not measured by the general weather station. To solve this, Lemke and Kjellstrom (2012) compared the available studies where WBGT had been modelled using the weather station data that is generally provided and found that the model developed by Liljegren et al. (2008) was most suitable for outdoor WBGT and the model developed by Bernard is for indoor WBGT.

Some problems are noted in the literature. Liljegren et al. (2008) discussed that the spatial differences at the measurement points seemed to affect the data more than the model error [66], indicating that the spatial difference plays a major role in WBGT assessment. Another problem discussed was the issue with irregular solar radiation, where one part of a site may by shaded by clouds whereas another may be fully irradiated by the sun. Such irregularities are both difficult to predict and to quantify. Lemke and Kjellstrom (2012) discussed that the error between the Liljegren method and measured values was smaller than errors measured using different instruments [16] signifying the feasibility of the approach. They also pointed out the slow response because of the use of a 15-cm globe in instruments gives rise to measurement errors in radiant temperature caused by rapid cloud cover changes (difficult to reach equilibrium). Estimates made for several Asian cities showed that afternoon WBGT can be 2–3 °C higher in the sun than in the shade stressing the challenges with accurate estimation of workplace microclimates [67].

WBGT is a well-accepted screening tool for heat stress but heat balance models have since then been developed to give a more accurate and specific prediction of heat balance and body responses by incorporating all thermal environment factors and individual data such as metabolic rate, body weight and height, the clothing worn, and thermal acclimatization. Where WBGT can provide the answer if there is a risk of heat stress or not, other models can provide detailed analytical determination of body heat storage and thermal physiological responses such as core temperature change and sweat rate (hydration requirement) allowing for specific advice. The latest edition of WBGT now incorporates the effects of clothing [68].

Individualization of the WBGT can be partly done by correcting reference values through clothing adjustment values, activity intensity, and heat acclimatization. However, WBGT does not predict thermo-physiological responses [68]. When combining several thermal models and indices to assess thermal stress, one needs to keep in mind that WBGT cannot be used for the screening of cold stress. The WBGT reference values are also corrected depending on the acclimatization status. According to the standard, an acclimatized person is one who has been exposed to the hot working conditions (or similar or more extreme conditions) for at least one full working week immediately prior to the assessment period. Other literature suggests that a person is considered fully acclimatized if core temperature reaches 38.5 °C for one hour per day for at least ten consecutive days [69]. Caution must be used when determining acclimatization as the usage of air conditioning and other mitigation strategies may impair the acclimatization progress. A person may also be partially acclimatized.

#### 4.1.2. Predicted Heat Strain (ISO 7933)

Predicted heat strain (PHS) is meant to predict the risk of heat strain during an 8-h work shift, providing a duration limit caused by the total water loss and core temperature rise [70]. The model is challenged by concepts such as intermittent breaks, protective clothing with high thermal insulation, and evaporative resistance which lowers the robustness of the model.

PHS suffers from a few limitations; the model is limited to 3 m/s wind speed, 0.1-1 clo basic clothing insulation [71,72] and the user should be cautious when assessing protective clothing [73]. PHS asks for posture which affects solar radiation incidence, ability to drink, and clothing properties. Acclimatization status is crucial as this affects the sweat capacity improving the heat loss capability, an acclimatized person is assumed to sweat 25% more [70].

Kampmann et al. (2012) compared the universal thermal climate index (UTCI) to WBGT and PHS. They observed an underestimation of rectal temperatures in PHS but a better sweat prediction by PHS compared to UTCI [74]. This underestimation was also supported in the study comparing PHS with the Fiala model [75] and in another study using water loss [76].

Lundgren et al. (2014) pointed out that the workers could already have been exposed to heat before the work shift started [77]. Many of the workers commuted to work, experienced elevated heat levels throughout the evening, night, and morning. Currently the PHS model assumes an initial core temperature value of 36.8 °C which may underestimate heat strain depending on the worker’s activity before arriving to the workplace.

PHS show steep increase when working again after a break. This limitation in dynamic behaviors has been reported in previous studies ([78]). This is one of the issues why the standard is now under revision [79]. It can be expected that in most scenarios, workers can take intermittent breaks for hydration and rest during exposure making such a limitation a problem for actual work assessment.

The model is limited to 3 m/s of air velocity which proves challenging as evaporation is the main source of heat loss during hot conditions. The water vapour gradient between the skin and the air is the driving force of evaporation; in order to maximize this heat flux dry air must replace the moist to prevent saturation of the air next to the skin.

Similar to WBGT, PHS is greatly influenced by the mean radiant temperature where microclimates can change quickly depending on the shade or reflective surfaces. Such uncertainty in microclimate must be taken into consideration when interpreting the model output data. The mean radiant temperature is estimated using the globe temperature measurement. Weihs et al. (2012) investigated the uncertainty of UTCI derived by uncertainties in predicting solar radiation using measured and modelled weather data, concluding that the worst case has an error of 3.0–5.9 K in the UTCI scale [80]. Another approach is using cloud-specific values for scattering [81] together with cloud cover data given by forecast data.

### 4.2. Cold Stress Model and Index

#### 4.2.1. Insulation REQuired (ISO 11079)

The Insulation REQuired (IREQ) model is a tool, based on the body heat balance to calculate the clothing insulation needed to maintain thermal comfort (IREQneutr) or insulation required to avoid adverse health effects but allow for slight cooling (IREQmin) compared to thermal comfort [82,83]. The model takes into account the metabolic rate of the user and the four thermal climate factors including air temperature, mean radiant temperature, humidity, and air velocity. The model will provide the user with required clothing insulation for IREQneutr and IREQmin, and duration limited exposure (DLE) based on the clothing insulation input (available insulation worn by the user). IREQ uses the basic insulation of the whole clothing ensemble for body heat balance calculation, hence it does not consider local insulation; however it recommends the use of clothing with evenly distributed insulation and proper protection of the extremities. Completely even distribution of insulation is rarely the case because of design, clothing layers, and body movements that displace the clothing system through creases and overlapping layers and the pumping effect [15].

As mentioned, IREQ is calculated based on and recommendations for the insulation of the whole body ensemble. It does not provide specifically local insulation required for the extremities. One major issue is if extremities are poorly insulated compared to the rest of the body to cause discomfort, pain, loss of finger dexterity, and extra local cooling [82]. A recently developed tool—The Cold Weather Ensemble Decision Aid (CoWEDA) based on the six cylinder thermoregulatory model provides the possibility to select gloves, footwear, and headgear to protect the extremities [84].

An investigation reported discrepancies between standard and official model code which could lead to a large error in DLE even though the IREQ difference was small [85], the code seems to provide better results than the standard and such discrepancies could be investigated in a standard revision. A field study using IREQ in the temperature range 0–15 °C proposed by using statistical analyses that the model is applicable up to 15 °C [50]. Further validation could initiate a standard revision to extend the temperature range of IREQ from +10 °C to +15 °C.

A challenge is the microclimate around the human body attributed to physical labor where variation in intensity will demand different clothing; however, it is quite rare that a person is able to change different clothing sets for different working tasks [50]. Using clothing that can be opened up or with removable pieces offers flexibility for various work tasks.

Similar to WBGT and PHS, solar radiation will affect the body heat balance calculated by IREQ as it warms up the person. During winter in polar and temperate climate zones, the ground emissivity plays an important part as the direct, diffuse, and reflective radiative fluxes are heavily influenced by cloud cover and the surface temperature. Similar to the effect of a carport keeping the car windows from freezing, shelters and particularly cloud cover will decrease the diffuse and reflective radiant temperature loss at the same time as the cloud cover shields direct solar radiation. On days with clear skies, the presence of the sun may be the thermal climate factor causing the difference of staying in thermal comfort and rapidly cooling down because of the loss of radiated heat when the sun goes down. The azimuth angle of the low standing sun also plays an important factor for the human body [15] in the polar latitudes. However, a challenge is that these parameters are not directly available from meteorological weather forecast data. There is a need to include these parameters into weather station measurements and forecast in terms of the direct effect of thermal climate on human thermal physiological responses, heat, and cold induced illnesses.

Initial cooling may start a series of responses in the body, such as shivering to increase metabolism and vasoconstriction to minimize the amount of dissipated heat. Cold exposure may cause hyperventilation followed by hypoventilation and at last an erratic breathing pattern [86]. Cardio- and cerebrovascular functions are impaired when the core temperature decreases with only a few degrees, mental abilities also become impaired [64].

#### 4.2.2. Wind Chill Temperature

IREQ does not use wind direction as input, it would however have an impact as the cooling effect of cold winds would especially be increased for naked skin if moving against the flow or decreased if travelling with the wind. The metabolic rate increase when moving against the wind and decrease with the wind in the back. This is considered in PHS by simply increasing the value of the air velocity as a function of the angle of the wind flow and could be integrated in IREQ using the same approach. The wind chill index is calculated by the air temperature and air velocity, and focuses on local skin convective cooling, making both variables crucial for proper evaluation of exposed skin where a decrease in wind chill index (<0 °C) deteriorates the manual hand performance and increases the risk of freezing injuries [65,84,87,88]. The wind chill temperature is categorized into several risk levels based on how quickly exposed skin is expected to freeze [89].

### 4.3. Indoor Thermal Comfort and Thermal Stress

#### Predicted Mean Vote and Predicted Percentage Dissatisfied (ISO 7730)

Predicted mean vote (PMV) is a model that uses the body heat balance equation to predict the thermal sensation at a given indoor environment condition on a scale +3 (Hot) to −3 (cold) [90]. In combination with PMV, the predicted percentage dissatisfied (PPD) is used to predict the percentage of a population that would be dissatisfied with a given thermal environment.

A critical assessment concluded that PMV is strongly affected by the measurement accuracy, meaning that slight errors in input may give unsatisfactory results. An error of 10% in clothing insulation and metabolic rate may result in a ±0.25 difference on the PMV scale [91]. Air velocity and radiant temperature affect the model significantly at lower metabolic rates. When assessing the uncertainties of all variables, the uncertainty of the model may become as great as ±1 on the PMV scale [91].

The indoor temperature may be significantly higher compared to the outdoor temperature. A difference of up to 50% is possible because of factors such as technology, room orientation, window placement and size, individual behavior and social systems (Lundgren 2019). As staying inside is a natural mechanism to shield oneself from outdoor exposure such as solar radiation or cold, improving indoor climate assessment is crucial to predict thermal stress. As seen in the study in China, one major challenge is to properly adapt buildings against climate variation [49]. Buildings have been our first thermal barrier to maintain a steady surrounding climate since man was first able to build shelters. With increasing extremes [1] and climate variability, naturally ventilated buildings must be able to both cool down and warm up residents depending on season and protect against the extremes. With poor construction and outdated adaptation capabilities, extremes may be intensified during heat waves and vulnerable individuals may be trapped in their ill-suited homes. This signifies thermal stress as a socio-economic problem where poverty or financial hardship put individuals at further risk. With more accurate indoor climate estimates, heat-health warning systems may provide more accurate alerts based on risk factors mentioned in this review. In order to get more accurate predictions, indoor air temperature, air velocity, humidity, and mean radiant temperature must be measured or estimated based on outdoor prevailing weather conditions and building characteristics for naturally ventilated buildings must be specified [92]. The importance of each of the thermal climate factors may be different in indoor environments compared to outdoor environments. If WBGT heat stress index and PMV model are used, all four thermal climate factors are taken into consideration for heat stress assessment indoors.

## 5. Individual Aspects

### 5.1. Fitness and Age

An individual factor to better cope with thermal stress is fitness, studies have shown that the capacity to cope with hotter environment increase with aerobic capacity [93]. Body size greatly influences heat balance as a larger body allows greater heat dissipation capacity thanks to more surface area, however obese people will find it more difficult to get rid of heat than leaner subjects [94]. With increasing age, maintaining thermal balance becomes more difficult as body composition, fitness, sweating capacity, cardiovascular function, and blood flow through the body deteriorates. Autonomic body responses to cold exposure such as vasoconstriction function reduce with age [95,96]. When aging, the ability to dissipate heat e.g., through sweat evaporation has been reported to decrease which increases the body heat storage if proper actions are not taken [97,98,99]. When comparing different age groups with similar fitness and body composition, the decrease in heat loss is less attributed to chronological age than individual fitness and body composition differences [100], the compromised ability to dissipate heat has been reported around 40 years of age in dry conditions [97]. Nakai et al. (1999) showed that in the age groups 5–64, there is a vast difference in mortality between the sexes. Most were men caused by physical exertion in heat because of sports activities and occupational exposure compared to infants and elderly where there was no difference [4].

### 5.2. Sexes

The differences between sexes in a heat balance perspective is a challenging topic. Studies have shown that anthropometric data and aerobic capacity (VO2_max_) render the variable of sex excessive for heat strain [101,102].

A field study performed by Lundgren et al. (2014) highlighted that female workers wore additional protection over their traditional clothing which out of a practical standpoint decreased the possibility to dissipate heat compared to their male counterpart [77]. When comparing PHS with the Fiala model against experimental data, both models predicted male subjects more accurately than female subjects indicating an overall need to validate models for women [76]. Iyoho et al. (2017) found that women in heat sweat less than males and shiver less during cold exposure. When accounting for these findings in their model the model output was more consistent with experiment data [103]. 

When analyzing the applicability of IREQ in 0–15 °C [50], Griefahn (2000) identified that women wore protective clothing with significantly lower clothing insulation compared to their male counterpart who matched the same workload and air temperature. This discrepancy was attributed to the physical difference between genders where women often have better physiological insulation provided by their subcutaneous fatty tissue [50]. However, a test performed in extreme cold indicated that females perceived more cooling even though they wore more insulation, this may be partly explained by different body mass and subcutaneous fat [104]. A thorough comparison of the differences of required clothing insulation between genders would be valuable to individualize cold stress warning and protection requirement against cold.

Hartgill et al. (2011) followed 15 pregnant women through their pregnancy and concluded that their core temperature decreases from the first trimester and considerably lowers the thermoneutral zone before stabilizing again three months after giving birth [105]. This difference further indicates the need to provide personalized assessment of thermal stress and advice of coping strategies.

The main concern when applying the available ISO standards for a wide population is that all models have been validated for fit, healthy male subjects. This calls for a great need to further study, validate, modify, or provide solutions to apply adjusted models for the rest of the population such as women, elderly, children, and people with disabilities or illnesses (chronic or acute).

### 5.3. Behavioral Aspects

There are several behavioral ways to alleviate thermal stress. In most cases a person is able to freely choose their clothing to suit the environment, add a layer or modify it by opening a button. Another way is to increase metabolic rate to stay warm or self-pace which was discussed by Miller (2011) to avoid generating too much internal heat. In extreme thermal conditions, a person may avoid direct solar radiation, increase ventilation, or use water cooling.

Inside a naturally ventilated building, a person may avoid sitting next to big windows when the sun is out, open up a window to force a draft or use electric fans to increase the local air velocity to increase evaporative cooling [106].

The issue with thermal stress is most obvious when people are not able to adapt to thermal stress by behavioral aspects. In certain jobs when working with machinery on an assembly line, it is not possible to self-pace to control metabolic rate. When suddenly caught off guard in a weather hazard or there are certain protective clothing requirements, the adjustment of clothing is limited [107].

## 6. Weather Forecast Data

An issue with using meteorological data as input is that it is impossible to properly predict microclimate since weather stations are positioned to be as unaffected by microclimate as possible. However, people will most certainly experience a microclimate designed by their work place, home or influenced by the natural surroundings. Wind speed will be strongly affected by topography and buildings, either by limiting the air movement around the individual or by exposing the location to increased turbulence. Most weather data are updated on an hourly basis introducing data input for the models with a low temporal resolution where errors can make microclimate predictions inaccurate. Therefore, it is necessary to continuously improve and extend meteorological service to provide customized higher resolution in e.g., urban settings [108]. If meteorological services would complement urban stations with urban microclimate models and local stations in workplaces, then such microclimate predictions could become more accurate and applicable if openly available.

An attempt to measure WBGT in Brazil by integrating weather station data showed that the approach is feasible with proper interpolation between the stations as long as the distance between them does not exceed 80 km [109]. In most cases the model used in Brazil and that by Liljegren (2008) seem to provide reliable results for Tg, Tnwb, and WBGT. However, mean radiant temperature is not directly available from meteorological data, neither from Liljegrens model. Mean radiant temperature is a necessary input for PHS, PMV, and IREQ models. Mean radiant temperature can be indirectly estimated using various models-based and weather station data, geographical locations, time of the day, day of the year, etc.

Weather stations measure wind speed at 10 m height whereas human thermal models use air velocity data near human body e.g., at 2 m height which is more relevant for the body heat exchange, since wind speed is a critical factor for convection and evaporation. The conversion from 10 m to 2 m wind speed data introduces an error. The conversion is based on surrounding ground type and seasonal variances, if not properly corrected it could introduce an error of 15–40% [110,111]. Properly measuring wind for microclimates for heat balance calculations will improve the accuracy of thermal stress prediction for everyday exposure for individuals heavily affected by the surroundings. The thermal climate factors needed in the human thermal models and data provided by weather stations are listed in Table 1.

## 7. ClimApp Case Study

The ongoing EU ClimApp project aims to translate climate service information into personalized warnings and adaptation strategies to cope with thermal stress (http://www.jpi-climate.eu/nl/25223441-ClimApp.html). It strives to cover a wide range of thermal environments from extremely hot to extremely cold climates by combining the thermal stress predictions using ISO standard based thermal models and indices. By widening the operational range and using more parameters, more challenges are introduced which needs validation. Every model and index make assumptions and simplifications in order to provide operational results in the range they are validated for. The vision of ClimApp is to be individualized, versatile, and educational by providing users with a supporting tool that will help to make decisions and minimize mishaps by poor judgement or inexperience. If microclimate data is available, then the user should be able to enter such data and receive feedback or warnings of his or her surroundings. Like any warning system, ClimApp relies on high accuracy in the weather forecast data, physical work intensity (metabolic rate), and clothing thermal insulation data. In order to prepare for extreme weather events, warnings must be provided in a timely manner as not all adaptation strategies can be planned last minute [117].

ClimApp may be applicable and complementary to the existing heat-health warning systems to offer further insight of whom the vulnerable groups are for both heat and cold. By creating arbitrary citizen profiles, warning systems can then identify these vulnerable groups more accurately and provide more detailed warnings and advice.

A study to assess the risks of heat exposure during the Olympic Games marathon in Tokyo 2020 was performed using mobile measurement equipment and thermal model to compare the changes in microclimate throughout the race [118]. The result and conclusions were that even during overcast weather, risk of heat stress is present. During the second half of the marathon, many stretches are in direct sunlight posing a higher risk because of the increased solar load. Similar findings could be obtained by ClimApp that could act as a supporting tool for planning sport and exercise events, particularly if high resolution urban microclimate data are measured or openly available through urban climate modelling [108].

## 8. Future Research Needs

Supported by this literature review, future research should address the validity of integrating human thermal models with weather forecast data to predict thermal stress and the continuity of different thermal models. A study should compare the differences between predicted thermal stress based on weather forecast with that based on real microclimate measurements. Different settings could be tested in order to validate and improve this approach. Models to predict urban microclimate and indoor climate based on weather forecast data should be further developed. Vulnerable groups should be a focus from the human thermal model perspective where adjustments and corrections may be required. In order to better predict the thermal stress, means to directly measure radiant temperature in weather stations or indirectly estimate mean radiant temperature should be investigated from the human thermal model point of view [112,113,114,115,116]. By implementing new approaches to predict the mean radiation temperature validation for each model could be done by testing the uncertainty in the model output similarly to what Weihs et al. (2012) did for UTCI [80].

## 9. Conclusions

In conclusion, integrating human thermal models with weather forecast data is feasible. It can extend meteorological services to better suit end users for different environmental challenges. It also makes the internationally established human thermal models and indices in the form of standards accessible by a wider population. The integration of meteorological data and human thermal models such as ClimApp can provide a tool for a wider population with personalized thermal stress warnings and advice when facing extreme weather events. Yet, great consideration must be used when applying the human thermal models. Weather stations are situated to be as unaffected by microclimate as possible, however individuals will most likely face thermal stressors in their specific microclimates. This indicates that this approach may be applicable as a screening tool for individuals rather than a case specific tool intended for local environments, as the input data from weather forecast may deviate from microclimate. If the deviation is big, the complement of measured microclimate data that is input by users may be necessary. The second challenge is the validity of applying the ISO thermal models to the general population as the models were developed with the data of healthy fit males. Further research on possible adjustments of the thermal models and indices to fit to vulnerable population is needed.

## Figures and Tables

**Table 1:** Available weather forecast data and required input parameters for thermal indices and body heat balance models. The estimated mean radiant temperature (Tmrt) for all models and indices are based on previous literature [112,113,114,115,116].

	Air Temperature (Ta)	Air Velocity (Va)	Relative Humidity (RH)	Cloud Coverage	Black Globe Temperature (Tg)	Natural Wet Bulb Temperature (Tnwb)	Mean Radiant Temperature (Tmrt)	Operational Range (Ta)	Sources of Data, Models and Indices
Weather forecast	√	√ (10 m)	√	√					ECMWF, NOAA, etc.
WBGT	√	√ (2 m) [68]	√		Estimated [66]	Estimated [66]	Estimated [68]	+10–60 °C (Ta sensor)	ISO 7243:2017
PHS	√	√ (2 m)	√				Estimated [70]	+15–50 °C	ISO 7933:2004
PMV	√ (indoor)	√ (indoor)	√ (indoor)				Estimated (indoor) [90]	+10–30 °C	ISO 7730:2005
IREQ	√	√ (2 m)	√				Estimated [83]	<10 °C	ISO 11079:2007
Wind Chill	√	√ (2 m)						<0 °C	Included in IREQ, Environment Canada [89]
